# Role of Chemerin and Perivascular Adipose Tissue Characteristics on Cardiovascular Risk Assessment by Arterial Stiffness Markers in Patients with Morbid Obesity

**DOI:** 10.3390/jcm12082885

**Published:** 2023-04-14

**Authors:** Viviana Aursulesei Onofrei, Ecaterina Anisie, Carmen Lacramioara Zamfir, Alexandr Ceasovschih, Mihai Constantin, Florin Mitu, Elena-Daniela Grigorescu, Antoneta Dacia Petroaie, Daniel Vasile Timofte

**Affiliations:** 1 Department of Medical Specialties, Grigore T. Popa, University of Medicine and Pharmacy, University Street No. 16, 700115 Iasi, Romania; 2 Cardiology Clinic, St. Spiridon, Clinical Emergency Hospital, Independence Boulevard No. 1, 700111 Iasi, Romania; 3 Clinical Rehabilitation Hospital, Cardiovascular Rehabilitation Clinic, Pantelimon Halipa Street No. 14, 700661 Iasi, Romania; 4 Academy of Medical Sciences, Ion C. Brătianu Boulevard No. 1, 030173 Bucharest, Romania; 5 Romanian Academy of Scientists, Dimitrie Mangeron Boulevard No. 433, 700050 Iasi, Romania

**Keywords:** arterial stiffness, adipokines, morbid obesity, cardiovascular risk, perivascular adipose tissue

## Abstract

Background and objective: The development of arterial stiffness (AS) in obesity is a multifactorial and complex process. The pleomorphic actions of adipokines and their local activity in perivascular adipose tissue (PVAT) are potential modulators of AS appearance and progression. We aimed to assess the correlations between two adipokines (chemerin, adiponectin), PVAT morphological changes (adipocyte size, blood vessel wall thickness) and AS parameters in the special subgroup of patients with morbid obesity. Material and methods: We enrolled 25 patients with morbid obesity and 25 non-obese patients, who were age- and gender-matched, untreated for cardiovascular risk factors, and admitted to hospital for laparoscopic surgical procedures (bariatric surgery for morbid obesity and non-inflammatory benign pathology surgery for non-obese patients). Before the surgical procedures, we evaluated demographic and anthropometric data and biochemical parameters including the studied adipokines. Arterial stiffness was evaluated using a Medexpert ArteriographTM TL2 device. In both groups, adipocyte size and vascular wall thickness as well as local adiponectin activity were analyzed in PVAT from intraoperative biopsies. Results: In our study, adiponectin (*p* = 0.0003), chemerin (*p* = 0.0001) and their ratio (*p* = 0.005) had statistically significant higher mean values in patients with morbid obesity compared to normal-weight patients. In patients with morbid obesity there were significant correlations between chemerin and AS parameters such as aortic pulse wave velocity (*p* = 0.006) and subendocardial viability index (*p* = 0.009). In the same group adipocyte size was significantly correlated with another AS parameter, namely, aortic systolic blood pressure (*p* = 0.030). In normal-weight patients, blood vessel wall thickness positively correlated with AS parameters such as brachial (*p* = 0.023) and aortic augmentation index (*p* = 0.023). An important finding was the negative adipoR1 and adipoR2 immunoexpression in PVAT adipocytes of patients with morbid obesity. Additionally, we found significant correlations between blood vessel wall thickness and blood fasting glucose (*p* < 0.05) in both groups. Conclusions: Chemerin and adipocyte size could be predictive biomarkers for AS in patients with morbid obesity. Given the small number of patients included, our results need further validation.

## 1. Introduction

### Potential Biomarkers for Assessing Cardiovascular Risk in Morbid Obesity

Cardiovascular disease (CVD) is one of the leading causes of mortality, accounting for about a third of deaths worldwide [1,2,3,4]. The increasing prevalence of major cardiovascular risk factors and the identification of new biomolecules involved in global cardiovascular risk assessment require the development of new, easy and clinician-friendly diagnostic algorithms to adequately estimate the cardiovascular outcomes [3,5,6]. Obesity is a major risk factor in the cardiovascular continuum, deeply implicated in the development of atherosclerotic processes [7,8,9]. Obesity, dyslipidemia, diabetes mellitus and hypertension are also involved in the progression of arteriosclerosis defined as arterial stiffness and atherosclerosis, thus, have a prognostic role. Literature data have correlated their presence with major molecular changes in adipose tissue metabolism [10,11]. Adiposity acts as an endocrine tissue that secretes adipokines, molecules involved in altering immune response, lipid metabolism, insulin resistance and angiogenesis [12,13,14,15,16]. 

Obesity leads to increased expression of pro-inflammatory adipokines and thus, maintains a constant inflammatory status [17], which contributes to the development of atherosclerotic CVD [14,18]. Chemerin is an adipokine that modulates metabolic changes and correlates with body mass index (BMI), insulin resistance and serum triglyceride levels [5,19]. The literature highlights its role as an independent predictor of coronary artery disease and acute coronary event risk [20,21,22]. Chemerin also acts at the perivascular tissue level; clinical studies in the literature show an association between its high local titers and the presence of both aortic and coronary atherosclerotic lesions [22,23]. There are data about the modulatory role of chemerin in the development of arterial stiffness through its inflammatory action and interaction with the components of metabolic syndrome [24,25,26]. The development of arterial stiffness in patients with obesity is a multifactorial process, mainly determined by inflammation of perivascular adipose tissue (PVAT), remodeling of the extracellular matrix, alteration of the immune system and development of cellular and endothelial stiffness in vessels [27,28].

Based on the known role of cytokines and some adipokines secreted by PVAT, various current clinical studies are designed to determine its potential as a therapeutic target to lower arterial stiffness and cardiovascular risk [29]. The influence of PVAT on vascular reactivity has been investigated since the 1990s. More recently, preclinical and clinical evidence have demonstrated that PVAT characteristics are associated with arterial stiffness [30,31,32]. Metabolic changes produced in adipose tissue (secondary to excessive secretion of TNF-α and adiponectin) amplify the pro-inflammatory status by activating a diverse cell population in the vascular wall and modulating insulin sensitivity and lipogenesis, mechanisms that contribute to arterial stiffness [27]. 

The aim of this study was to evaluate the potential role of adipokines such as chemerin and adiponectin and PVAT morphological characteristics as biomarkers associated with arterial stiffness in patients with morbid obesity, given its role of independent cardiovascular risk factor.

## 2. Materials and Methods 

### 2.1. Study Design and Population

We conducted a case-control study which included 25 consecutive adult patients with morbid obesity (body mass index [BMI] > 40 kg/m^2^) and 25 age- and gender-matched non-obese patients who were part of the control group (BMI < 30 kg/m^2^). The study period was January–May 2017. All patients were referred to the Third General Surgery Department of “St. Spiridon’’ Hospital for laparoscopic surgical procedures (bariatric surgery for patients with morbid obesity and non-inflammatory benign pathology for normal-weight patients). Cardiological evaluation was part of preoperative assessments. Patients with any treated cardiovascular risk factors, medical or surgical comorbidities generating inflammation, or treated with drugs interfering inflammation were excluded. Also, patients who presented with any three of the five criteria for defining metabolic syndrome [33] or had concurrent enrollment in another study were excluded. Patients included in the study had no associated cardiovascular risk factors requiring drug treatment.

### 2.2. Laboratory Measurements 

We evaluated demographic parameters (age, gender); cardiovascular (CV) risk factors; anthropometric parameters; vital signs (systolic blood pressure (SBP, mmHg), diastolic blood pressure (DBP, mmHg)); biological parameters (both biochemical and hematological); as well as arterial stiffness (AS) parameters. Anthropometric measurements included BMI (kg/m^2^), waist circumference, waist to hip circumference ratio (WHR) and index of central obesity (waist circumference to height ratio). 

As we described in a previously published paper [34], all venous blood samples were collected after overnight fast and processed using specific techniques. The assessment of biochemical parameters was performed within two hours. Fasting glucose, total cholesterol and triglycerides were determined applying enzymatic colorimetric method, while HDL-cholesterol was measured using imunoturbidimetry. LDL-cholesterol levels were calculated using the Friedewald equation [35]. Fasting insulinemia and serum TNF-α were assessed using chemiluminiscence immunoassay kits (Siemens Healthcare GmbH., Erlangen, Germany) automated by an Immulite 1000 analyzer. Insulin resistance (IR) and insulin sensitivity (IS) completed the assessment of metabolic profile and were calculated applying the Homeostasis Model Assessment (HOMA) and quantitative check index (QUICKI) [36], respectively. 

Serum levels of the two studied adipokines (chemerin and adiponectin) were processed after venous blood collection in vacutainer tubes without anticoagulant and centrifugation. Chemerin, known as retinoic acid receptor responder protein 2—RARRES2, is a 14 kDa protein, 131–137 amino acids long, resulting from proteolytic cleavage of the inactive molecule [37]. Serum levels of chemerin and adiponectin were assessed using quantitative specific human ELISA (enzyme-linked immunosorbent assay) kits (ab155430, ab99968, respectively) supplied by Abcam Cambridge, UK, for research use only. Chemerin/adiponectin ratio was calculated for studying the relation with AS. All biological samples were stored at −20 °C and processed after completing the enrollment of patients [37]. 

### 2.3. Arterial Stiffness Evaluation 

Arterial stiffness was assessed by oscillometric method using the Medexpert ArteriographTM TL2 device. After data entry into the software (identification data, anthropometric parameters, age) and brachial blood pressure (BP) measurement, the AS parameters were generated: aortic pulse wave velocity (PWV), aortic and brachial augmentation index (Aix), systolic area under the pulse wave curve (SAI) and diastolic area below the pulse wave curve (DAI), diastolic reflection area (DRA), central BP and central pulse pressure (PP). The recommendations of the 2012 Expert consensus document [38] were followed. A complete report was produced in approximately 10 min. Subendocardial viability ratio (SEVR) was determined using the ratio of the areas of the systolic and diastolic portions below the aortic pulse wave curve and denoted as systolic area index (SAI) and diastolic area index (DAI), respectively. Knowing that SAI is calculated as the product of mean systolic LV pressure and systole duration, and DAI as the product of the difference between mean aortic diastolic pressure and mean diastolic LV pressure and diastole duration, we rewrote the calculation formula as follows:

$$SEVR=\frac{\left(mean\;aortic\;diastolic\;pressure-mean\;diastolic\;LV\;pressure\right)\times diastole\;duration\;}{mean\;systolic\;LV\;pressure\times systole\;duration\;}$$

The measurement protocol using the Arteriograph device involved the following steps: (1) recording general patient data (name, date of birth, weight, height, arm circumference and abdominal circumference, the distance between the sternal notch and the upper edge of the pubic symphysis, without following the abdominal relief); (2) locating the area of maximum pulsatility of the brachial artery and positioning the device cuff at this level; (3) the initiation of measurements, with the patient lying on his back and tracking the recording of pulse waves on the monitor to observe the morphology of the route; and (4) the interpretation of the results. In addition, before and during the recording the examination was performed in a quiet environment; avoidance of speech and mobilization during the measurement were encouraged. When white coat hypertension was suspected, an attempt was made to reassure the patient and the measurement was repeated. Smoking and coffee consumption were suppressed at least 3 h before the examination, copious meals were avoided during this period, as was the administration of nitrates. Alcohol consumption was prohibited for a period of 10 h before AS measurements [39].

### 2.4. Local Adipocytes and Adiponectin Expression Evaluation

During laparoscopic bariatric surgery, intraoperative biopsy of abdominal perivascular fat (PVAT) was performed. Specimens of 1 cm^3^ were collected from the great/small epiploon region, including a visible small artery, for local adiponectin activity assessment. 

Samples from both groups were collected, fixed in 4% formaldehyde solution, embedded in paraffin, cut in 2 μm sections, stained (hematoxylin-eosin staining—H&E) and examined with a Nikon Eclipse 50i microscope. The sections were also subjected to immunohistochemical assay and treated with anti-Adiponectin Receptor 1/ADIPOR1 antibody EPR6626 (ab126611) and anti-Adiponectin Receptor 2/ADIPOR2 antibody (ab77612), according to standard protocols. Images were captured and analyzed using Zeiss Observer Z1 Tissue FAXS 4.2 Cell analysis SystemAdipocyte; size and number per field were quantified on microphotographs. Examination of the sampled sections included separate analyses of five different fields on slides obtained from the two batches. Initially, the long and short axis of the adipocytes were determined in order to make an assessment of the order of cell size in the two groups. The adipocytes detected in the center and the four extremities of each image were morphologically characterized. Subsequently, histological changes of the vascular wall layers were analyzed and the thickness of vascular wall was measured. The fields selected for analysis were chosen from the four extremities of each histopathological image so as to include vessels with the most different caliber and wall profile. We added a scale bar for all the images, to permit comparisons between control and obese groups.

### 2.5. Statistical Analysis

Statistical analyses were performed using SPSS statistics software (Statistical Package for the Social Science version 26, for Windows; SPSS Inc., Chicago, IL, USA). Initially, the descriptive analysis of the variables was performed for the continuous type variables, calculating the mean, median, minimum and maximum values, quartiles and standard deviation. Skewness (measuring the symmetry of the variables with respect to the mean value) and kurtosis (flattening coefficient) were determined to assess the normal distribution of continuous variables.

To compare the mean values between two groups of continuous values in order to identify statistically significant differences, the t test (independent *t* test) and ANOVA (one way analysis of variance) were used. Pearson and Spearman (r) correlation coefficients were used to assess the presence of correlations between the studied variables. A natural logarithmic transformation was performed for the variables without a normal distribution. This transformation allowed us to use the Pearson coefficient to determine if there was a linear correlation between the studied variables. Kendall’s tau coefficient was used to evaluate correlations in the whole sample. For the subsequent analysis of the relationship between variables that met the threshold for statistically significant correlations, a simple linear regression was performed. After selecting several independent variables that influenced a dependent variable, a simple linear regression was extended to a multiple regression. A *p*-value ≤0.05 was considered statistically significant.

### 2.6. Ethics

The study protocol was approved by the local Ethics Committees of “Grigore T. Popa” University of Medicine and Pharmacy Iasi and of “St. Spiridon” Clinical Emergency Hospital Iasi, and was conducted in accordance with the terms of the Helsinki Declaration. All participants signed an informed written consent before enrollment.

## 3. Results

Our study included 50 patients divided into two equal groups according to the presence of morbid obesity. Statistical analyses included several demographic, anthropometric and paraclinical parameters, which are presented in Table 1. Both groups included predominantly female patients (68% vs. 84%), with a slightly higher average age in the group of normal-weight patients (*p* = 0.021).

Data on risk factors were included among the data on clinical characteristics of the patients that were collected for the study. In our data, smoking was a more frequent cardiovascular risk factor in normal-weight patients, while dyslipidemia was more frequently encountered in patients with morbid obesity.

Patients with morbid obesity were associated with higher mean systolic (118.04 ± 11.72 mmHg vs. 129.36 ± 13.03 mmHg, *p* = 0.002) and diastolic blood pressure values (67.08 ± 7.89 mmHg vs. 75.28 ± 11.12 mmHg, *p* = 0.004), and pulse pressure values (51.32 ± 9.98 mmHg vs. 59.08 ± 11.28 mmHg, *p* = 0.013). Various metabolic disorders, such as changes in lipid and carbohydrate profile parameters were also observed. Although there were no statistically significant differences between the two groups, the mean serum values of total cholesterol (*p* = 0.718), LDL-cholesterol (*p* = 0.76), VLDL-cholesterol (*p* = 0.919) and triglycerides (*p* = 0.86) were higher in patients with morbid obesity. Patients with morbid obesity had statistically significant higher mean serum values of fasting glucose (*p* = 0.002) and uric acid (*p* = 0.006) as well as parameters associated with insulin metabolism (insulinemia, insulin sensitivity and insulin resistance) (*p* < 0.05). Mean serum values of adiponectin (*p* = 0.0003), chemerin (*p* = 0.0001) and their ratio (*p* = 0.005), as markers of adiposity, were also significantly different between the two groups. All patients enrolled in the study were evaluated for arterial stiffness, but there were no statistically significant differences between the two studied groups. 

**Figure 1:**
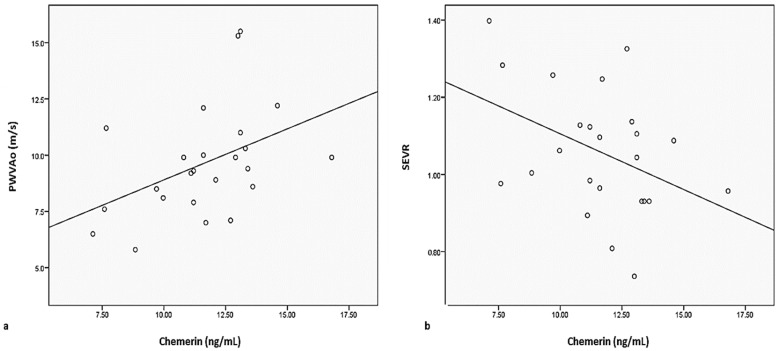
Correlations between serum chemerin levels and (**a**) PWVAo or (**b**) SEVR in patients with morbid obesity (PWVAo: aortic pulse wave velocity; SEVR: subendocardial viability index).

Among the studied arterial stiffness parameters, statistically significant correlations between serum levels of chemerin and PWVAo (r = 0.272, *p* = 0.006) or SEVR (r = −0.259, *p* = 0.009) were observed in patients with morbid obesity (Figure 1). 

In addition to the descriptive statistical analysis presented above, a series of tests for correlation between histopathological and paraclinical parameters were performed and results are presented in Table 2. 

In normal-weight patients, blood vessel wall thickness was significantly correlated with serum TNF-α levels (*p =* 0.013), fasting glucose (*p =* 0.049), age (*p =* 0.020) and with AS parameters such as brachial AIx (*p =* 0.023), aortic AIx (*p =* 0.023) and DRA (*p =* 0.044). In patients with morbid obesity, adipocyte size was correlated with serum VLDL-cholesterol (*p* = 0.001) and triglyceride levels (*p* = 0.001) as biological parameters, and with aortic SBP (*p* = 0.030) as arterial stiffness parameter. As in normal-weight patients, blood vessel wall thickness was significantly correlated with blood glucose (*p* = 0.035) in patients with morbid obesity.

### Histopathological Study 

Histological and immunohistochemical evaluation of white adipose tissue samples of patients from both groups was performed. 

Analyzing separately five different fields on the slides obtained from the two studied groups, we first determined the long axis of adipocytes to assess the order of size of fat cells in the two groups. Adipocytes detected in the center and at the four extremities of each image were monitored. Statistical processing of these data allowed us to reveal that adipocytes in the group with morbid obesity are more voluminous compared to those in the control group, suggesting that the abundance of adipose tissue may be the result of, not only increased adipocyte number, but also adipocyte hypertrophy. 

The second parameter assessed was blood vessel wall thickness. The fields selected for analysis were chosen from the four extremities of each image so as to include vessels with the most different caliber and wall profile. After completing the database, the statistical processing of data allowed us to observe that in the group with morbid obesity the blood vessels had a larger diameter and a thicker wall compared to the control group.

**Figure 2:**
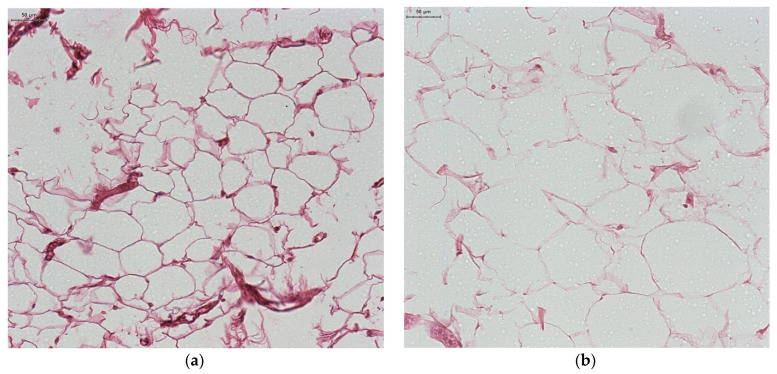
Adipose tissue: a control group ((**a**) non-obese patient); (**b**) Obese patient (H&E staining).

**Figure 3:**
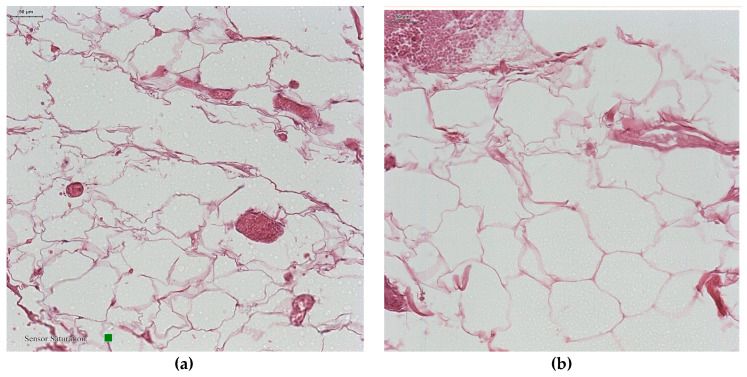
Histopathological images. Blood vessels, adipose tissue. (**a**) control group (non-obese patient); (**b**) obese patient (H&E staining).

Microscopic exam of samples from the control group revealed a normal morphology of white adipocytes, with a single lipid inclusion occupying almost the entire cytoplasm and a flattened nucleus compressed at one of the cell poles (Figure 2a). The histologic exam of samples from the group with morbid obesity revealed white adipocytes whose morphology did not differ significantly under light microscopy compared to that of adipocytes from the control group. However, their size was greater and in some areas the shape was slightly irregular, due to their adjacent compression (Figure 2b). A rich vascularization was mainly represented by microvascular elements; in our study, among the adipocytes, in the compact adipose tissue, small blood vessels with intact endothelium and vascular wall could be observed (Figure 3a). The vessels showed a consistent vascular wall, the tunica media was visibly thickened due to hyperplasia of smooth muscle fibers, and congestive areas were frequent. Most blood vessels were distended, with a stellate, irregular lumen and a sinuous periadipocytic course (Figure 3b).

**Figure 4:**
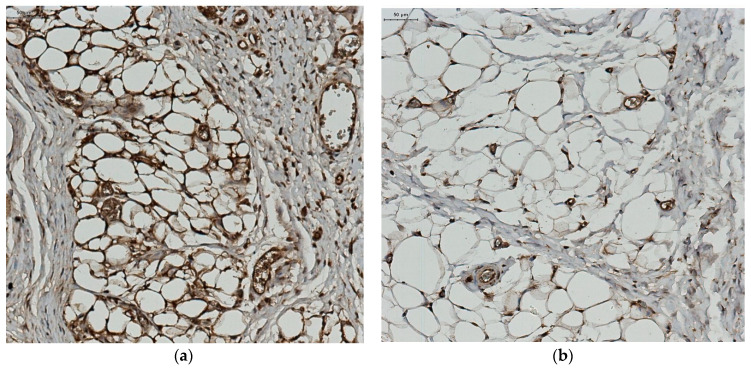
AdipoR1 immunoexpression (**a**) control group. AdipoR1—intense immunoexpression; (**b**) obese patient—AdipoR1 reduced immunoexpression.

**Figure 5:**
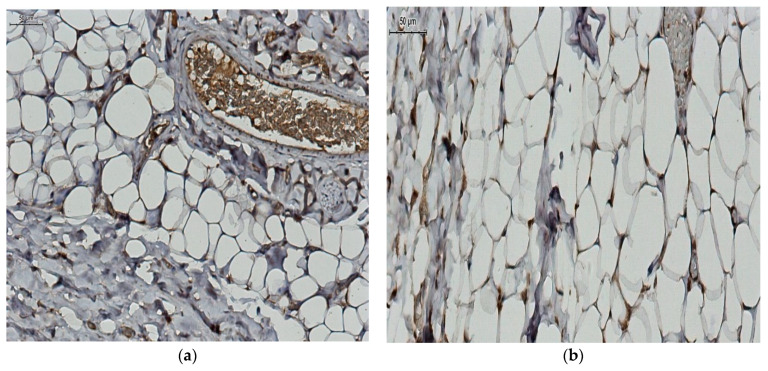
AdipoR2 immunoexpression (**a**) control group—AdipoR2 positive immunoexpression, (**b**) obese patient—AdipoR2 very low immunoexpression.

Immunohistochemical evaluation of the expression of the two adiponectin receptors (R1 and R2) was performed. We observed a positive AdipoR1 immunoexpression for adipocytes from the control group and a reduced, even negative AdipoR1 immunoexpression for the adipocytes from the group with morbid obesity (Figure 4). At the same time, we observed a positive AdipoR2 immunoexpression for adipocytes from control group and a very low AdipoR2 immunoexpression for the group with morbid obesity (Figure 5).

## 4. Discussion 

Current data in the literature suggest that adipokines have a pleomorphic action responsible for arterial stiffness development. So, we studied the potential roles of circulating chemerin and local adiponectin activity in PVAT, as well as morphological features of abdominal PVAT in relation to arterial stiffness and with demographic, anthropometric and metabolic parameters in a special subgroup of patients with morbid obesity.

In our study, patients with morbid obesity had higher serum values of lipid and carbohydrate profile parameters (*p* = 0.002 for fasting glucose). Insulinemia (*p* = 0.0004), insulin sensitivity (*p* = 0.0001) and insulin resistance (*p* = 0.001), as well as the mean serum levels of adiponectin (*p* = 0.0003), chemerin (*p* = 0.0001) and their ratio (*p* = 0.005) were parameters with statistically significant higher mean values in this group. 

In addition, our study patients with morbid obesity had higher, but normal mean blood pressure values compared to normal-weight patients. This observation is important as hypertension modulates the progression of arterial stiffness even in normal-weight patients. In this category of patients, Li et al. [39] demonstrated a significant correlation between visceral adiposity index and brachial PWV. In a similar study, Kim et al. [40] highlighted that brachial PWV correlates positively with WHR and visceral fat area, but not with BMI or abdominal circumference.

Yoo et al. [24] demonstrated that chemerin is an independent predictor for arterial stiffness, with multiple statistically significant correlations with both anthropometric and biochemical parameters. Similar correlations between chemerin and aortic PWV (*p* = 0.006) and SEVR (*p* = 0.009) were reported in our study among patients with morbid obesity. SEVR, also known as the Buckberg index, is an arterial stiffness parameter correlated with coronary flow reserve, useful in assessing coronary microvascular circulation. SEVR is a measure of cardiac oxygen supply-to-demand ratio that can be estimated by noninvasive validated methods [41], and is currently used in research and clinical practice [42]. The current data highlight the inverse relationship between decreased values of SEVR and cardiovascular risk [42]. In a recent study, Tocci et al. [43,44], demonstrated that overweight adolescent patients have reduced SEVR values compared to normal-weight patients, similar to their carotid-femoral pulse wave velocity and aortic systolic blood pressure. Other recent studies conclude that SEVR values decrease with increased number of metabolic syndrome elements (*p* = 0.005) [44] and are correlated with both IR and IS [45,46]. So, we could hypothesize that chemerin may be a biomarker for arterial stiffness and for the risk of microvascular coronary damage. 

Chemerin is also a determinant of endothelial dysfunction, with high titers negatively correlated with flow-mediated dilatation (odds ratio of 1.58) and arterial stiffness (odds ratio of 3.75) in hypertensive patients [47]. Our results suggest that in morbid obesity, chemerin would indicate the development of AS before hypertension is documented. Clinical studies also report that high serum levels of chemerin are associated with enhanced carotid intima media thickness (*p* = 0.035), and are a marker of subclinical atherosclerosis in diabetic patients [48]. In our group with morbid obesity and normal values of fasting glucose, chemerin was correlated with AS, also a marker of subclinical atherosclerosis. It is worth mentioning that this group had changes of insulin metabolism which could mediate chemerin action.

In peripheral tissues, chemerin induces tissue enlargement, while stimulating inflammation and angiogenesis in adipose tissue [11,49]. Obesity is morphopathologically characterized by hyperplasia and hypertrophy of adipocytes, accompanied by macrophage infiltration of adipose tissue [50]. These aspects correlate with pro-inflammatory status [50,51], as our study also demonstrated. Unlike the data from the literature, our study could not demonstrate statistically significant correlations between chemerin, chemerin/adiponectin ratio and histopathological parameters in perivascular tissues. However, our results suggest that adipocyte size is correlated with aortic SBP (*p* = 0.030), a parameter of AS, in patients with morbid obesity. Similar to our results, Arner et al. [52] demonstrated that PWVAo correlated positively with subcutaneous adipocyte volume and negatively with fat cell count in patients with obesity. Weight loss could be associated with improvement in PWVAo but positive long-term results on arterial stiffness can only be achieved by bariatric surgery [53]. Regarding blood vessel wall thickness, no significant correlations with parameters of arterial stiffness were reported in our study. These data are supported by the results of previously published clinical studies that showed arterial stiffness assessed by PWV measurement is independent of arterial wall thickening [54]. 

Knowledge regarding the relationship between PVAT and arterial stiffness among those with obesity is limited, and many confounders affect the ability to infer causality [55].

White visceral fat has an essential role in the production of adipokines, while PVAT through direct contact with blood vessels has a paracrine role, modulating cardiovascular effects, independent of obesity [56]. On the other hand, obesity could cause PVAT dysfunction, an emerging paradigm, thus, highlighting its deleterious effects. PVAT dysfunction is induced by complex and not fully elucidated interactions among adipocyte hypoxia, insulin resistance, oxidative stress, vascular inflammation, and macrophage activation in the early stages of atherosclerosis [57,58,59]. Clinical studies in the literature to date have demonstrated that PVAT infiltration with pro-inflammatory cytokines is a morphopathological feature in patients with obesity and insulin resistance, thus, contributing to the maintenance of inflammatory status, endothelial dysfunction and further development of arterial stiffness [27,60,61,62].

Studying different adipokines that could regulate the atherosclerotic process might provide new opportunities for developing potential biomarkers predictive for AS, which is especially important in apparently healthy obese individuals. Clarifying the relationship between adipokines and established markers of atherosclerosis is an effective approach to refine the characterization of cardiovascular risk in patients with obesity and prevent CVD [50]. Growing evidence suggest that, in obesity, some adipokines directly mediate the process of atherosclerosis, regulating the redox state and inflammation [63,64,65]. Very recently it has also been shown that humans with obesity have a higher expression of vascular/perivascular TNF-α [66], in addition to NF-Κb and IL-6. In our study, there was no statistically significant difference in the serum levels of TNF-α between the two groups, possibly due to the metabolic status of patients with morbid obesity.

Compromised bioavailability of adiponectin also has been established as an independent risk factor for type 2 diabetes and cardiovascular diseases [67]. Some studies link circulating adiponectin to vascular structural changes involved in early vascular aging, while locally produced molecules modulate the contractile function of small arteries [68,69]. 

Mean serum adiponectin levels were significantly higher among morbidly obese patients (*p* = 0.0003), a finding which is distinct from other published data and could suggest the presence of adiponectin resistance. Histopathological studies demonstrated that serum adiponectin levels were negatively correlated with adipose tissue mass. Low adiponectin titers correlate with the development of insulin resistance, type 2 diabetes or metabolic syndrome [70,71,72]. Several clinical studies demonstrated that serum adiponectin level is an independent predictor of aortic or brachial PWV [73,74]. This adipokine exerts its effects via two receptors, R1 and R2, whose expression is decreased in patients with obesity induced in experimental studies [75]. A high level of AdipoR1 is found in normal adipose tissue, while in obese patients its level is significantly reduced. In our study, the AdipoR1 and adipoR2 immunoexpression was clearly positive in normal adipose tissue. The negative results in patients with morbid obesity could be interpreted as difficult to detect because of the absence, or very low level of adiponectin receptors 

Our study has some limitations. Firstly, the most important one is the small number of patients included; our results could be seen as preliminary results which need further studies for validation. Secondly, it would have been preferable that patients with morbid obesity to be their own witnesses over a period of 6–12 months. Such a design involves some ethical limitations because it would be required a repeated biopsy and a longer observational period for study. Thirdly, we proposed the study of local adiponectin activity from abdominal PVAT, under the assumption that it shares similar properties with other regions of perivascular fat. 

## 5. Conclusions

The studied adipokine chemerin and adipocyte size in abdominal PVAT could represent predictive biomarkers for arterial stiffness in patients with morbid obesity. Although our study did not demonstrate the relationship between adiponectin and parameters of AS, the negative/weak immunoexpression of its receptors in abdominal PVAT could influence this process. In addition, our results could be influenced by the metabolic status of the patients studied. Given the small size of our sample, the current results need further validation. 

## Figures and Tables

**Table 1:** Demographics, anthropometric and paraclinical parameters in the two studied groups.

	Non-Obese Patients (n = 25)	Obese Patients (n = 25)	*p*
**Demographics and anthropometric parameters**			
Age (y)	43.36 ± 13.9	39.24 ± 8.74	0.021
Female sex (%)	17 (68%)	21 (84%)	-
BMI (kg/m^2^)	24.24 ± 3.15	43.9 ± 6.07	0.0001
Waist circumference (cm)	83.04 ± 8.75	125.5 ± 18.68	0.0001
WHR	0.83 ± 0.08	0.96 ± 0.10	0.0001
Index of central obesity	0.50 ± 0.06	0.75 ± 0.08	0.0001
Systolic blood pressure (mmHg)	118.04 ± 11.72	129.36 ± 13.03	0.002
Diastolic blood pressure (mmHg)	67.08 ± 7.89	75.28 ± 11.12	0.004
Pulse pressure (mmHg)	51.32 ± 9.98	59.08 ± 11.28	0.013
**Biological parameters**			
Total cholesterol (mg/dL)	197.80 ± 41.39	201.4 ± 27.17	0.718
HDL-cholesterol (mg/dL)	50.36 ± 14.94	50 ± 9.98	0.92
LDL-cholesteol (mg/dL)	125.04 ± 39.97	127.68 ± 23.48	0.76
VLDL-cholesterol (mg/dL)	24.50 ± 10.13	24.86 ± 14.39	0.919
Triglycerides (mg/dL)	121.24 ± 25.74	124.32 ± 17.96	0.86
Fasting glucose (mg/dL)	88.32 ± 8.80	99.28 ± 14.62	0.002
Insulinemia (µU/mL)	8.23 ± 7.98	24.47 ± 6.16	0.0004
Insulin sensitivity index *	1.82 ± 1.87	6.45 ± 3.73	0.0001
Insulin resistance (HOMA) (M/mU/L)	0.16 ± 0.12	0.13 ± 0.02	0.001
Uric acid (mg/dL)	5.29 ± 1.48	6.79 ± 2.19	0.006
TNF-ɑ (pg/mL)	11.34 ± 11.42	7.49 ± 3.38	0.116
Adiponectine (ng/mL)	16.36 ± 1.49	18.05 ±1.155	0.0003
Chemerin (ng/mL)	9.10 ± 1.89	12.22 ± 3.80	0.0001
Adiponectin/chemerin ratio	0.55 ± 0.12	0.67 ± 0.18	0.005
**Arterial stiffness parameters**			
Aortic Aix (%)	35.1 ± 16.2	24.1 ± 12.1	0.090
Brachial Aix (%)	−5.5 ± 0.32	−26.7 ± 0.24	0.110
Aortic SBP (mmHg)	119.42 ± 20.18	128.74 ± 20.81	0.114
Aortic PP (mmHg)	50.66 ± 12.69	52.26 ± 10.76	0.633
DRA	45.32 ± 18.82	49.68 ± 11.38	0.321
SAI (%)	46.41 ± 6.36	48.82 ± 3.81	0.112
DAI (%)	53.61 ± 6.06	51.28 ± 3.80	0.111
SEVR	1.19 ± 0.28	1.06 ± 0.16	0.054
PWVAo (m/s)	8.92 ± 2.14	9.59 ± 2.38	0.305
**Cardiovascular risk factors**			
Smoking	10 (40.0%)	9 (36.0%)	-
Fasting glucose above 100 mg/dL	-	2 (8.0%)	-
Dyslipidemia	9 (36.0%)	11 (44.0%)	-
**Perivascular adipose tissue parameters**			
Adipocyte size (µm)	6.62 ± 1.78	9.34 ± 2.11	0.027
Blood vessel wall thickness (µm)	6.92 ± 1.48	8.79 ± 2.12	0.0001

All values are expressed as mean ± standard deviation (SD) or n (%); y: years; BMI: body mass index; WHR: waist to hip ratio; HDL: high-density lipoprotein; LDL: low-density lipoprotein; SBP: systolic blood pressure; DBP: diastolic blood pressure; MBP: mean blood pressure; PP: pulse pressure; DRA: diastolic refelction area; SAI: systolic area under the pulse wave curve; DAI: diastolic area under the pulse wave; PWVAo: aortic pulse wave velocity; AIx: augmentation index; SEVR: subendocardial viability ratio; * We used the quantitative insulin-sensitivity check index (QUICKI).

**Table 2:** Correlations between adipocyte size and blood vessel wall thickness with demographic, clinical and paraclinical parameters.

	Non-Obese Patients				Patients with Morbid Obesity			
Parameters	Adipocyte Size		Blood Vessel Wall Thickness		Adipocyte Size		Blood Vessel Wall Thickness	
	r	*p*	r	*p*	r	*p*	r	*p*
**Biochemistry**								
Adiponectin	−0.220	0.123	−0.037	0.797	0.017	0.907	0.067	0.640
Chemerin	0.110	0.441	−0.189	0.190	−0.066	0.655	0.179	0.224
Chemerin/Adiponectin ratio	0.160	0.262	−0.124	0.387	−0.047	0.747	0.174	0.234
TNF-α	0.057	0.691	−0.357	0.013	0.074	0.607	0.104	0.469
Serum fibrinogen	−0.073	0.607	0.030	0.833	0.117	0.413	−0.094	0.513
HOMA	−0.093	0.513	−0.165	0.252	0.060	0.674	0.237	0.097
Insulinemia	−0.127	0.375	−0.171	0.233	0.043	0.761	0.193	0.176
Insulin sensitivity index	0.093	0.513	0.165	0.252	−0.060	0.674	−0.237	0.097
Fasting glucose	−0.034	0.815	0.287	0.049	−0.017	0.907	0.304	0.035
Total cholesterol	−0.144	0.427	0.185	0.198	0.181	0.207	−0.050	0.726
LDL-cholesterol	0.010	0.944	0.182	0.206	−0.044	0.761	0.007	0.963
HDL-cholesterol	−0.081	0.574	0.027	0.851	−0.176	0.223	0.010	0.944
VLDL-cholesterol	−0.037	0.797	0.114	0.426	0.482	0.001	−0.017	0.907
Triglycerides	0.003	0.981	0.101	0.483	0.482	0.001	−0.017	0.907
Uric acid	0.071	0.623	−0.082	0.574	−0.047	0.744	0.003	0.981
Serum creatinine	0.081	0.574	0.038	0.796	0.125	0.387	0.222	0.123
**Hemodynamic parameters**								
Systolic blood pressure	0.030	0.833	0.116	0.426	−0.269	0.061	0.239	0.097
Diastolic blood pressure	0.013	0.925	0.051	0.725	−0.212	0.141	0.183	0.206
Aortic pulse pressure	−0.088	0.543	0.126	0.386	−0.132	0.361	0.183	0.206
Mean blood pressure	0.020	0.888	0.132	0.361	−0.266	0.064	0.162	0.261
**Demographics and anthropometric parameters**								
Body mass index	0.153	0.283	−0.097	0.498	−0.124	0.387	0.093	0.513
Abdominal circumference	0.181	0.214	−0.124	0.398	0.003	0.981	0.203	0.160
Waist to hip ratio	−0.060	0.674	−0.138	0.337	0.170	0.233	0.193	0.176
Central obesity index	0.213	0.135	−0.252	0.079	−0.017	0.907	0.247	0.084
Age	−0.115	0.426	0.337	0.020	−0.249	0.087	0.061	0.673
**Arterial stiffness parameters**								
AIx brachial	−0.013	0.926	0.326	0.023	−0.043	0.761	0.180	0.207
SBP aortic	0.100	0.483	0.185	0.198	−0.311	0.030	0.220	0.123
PP aortic	−0.033	0.815	0.216	0.134	−0.281	0.050	0.230	0.107
AIx aortic	−0.033	0.515	0.326	0.023	−0.043	0.761	0.180	0.207
DRA	0.037	0.797	−0.292	0.044	0.261	0.071	−0.068	0.639
SAI	0.03	0.761	0.054	0.708	−0.242	0.092	0.117	0.413
DAI	−0.050	0.726	−0.094	0.512	0.259	0.072	−0.141	0.326
SEVR	−0.070	0.624	−0.067	0.640	0.248	0.084	−0.124	0.387
PWVAo	0.003	0.981	0.175	0.224	−0.151	0.293	0.054	0.708

r: Pearson Correlation; HDL: high-density lipoprotein; LDL: low-density lipoprotein; VLDL: very-low-density lipoprotein cholesterol; SBP: systolic blood pressure; DBP: diastolic blood pressure; MBP: mean blood pressure; PP: pulse pressure; DRA: diastolic reflection area; SAI: systolic area under the pulse wave curve; DAI: diastolic area under the pulse wave; PWVAo: pulse wave velocity at the central level; AIx: augmentation index; SEVR: subendocardial viability index;.

## Data Availability

The data presented in this study are available on request from the corresponding author. The data are not publicly available due to local policies.
